# Canagliflozin extends life span in genetically heterogeneous male but not female mice

**DOI:** 10.1172/jci.insight.140019

**Published:** 2020-11-05

**Authors:** Richard A. Miller, David E. Harrison, David B. Allison, Molly Bogue, Lucas Debarba, Vivian Diaz, Elizabeth Fernandez, Andrzej Galecki, W. Timothy Garvey, Hashan Jayarathne, Navasuja Kumar, Martin A. Javors, Warren C. Ladiges, Francesca Macchiarini, James Nelson, Peter Reifsnyder, Nadia A. Rosenthal, Marianna Sadagurski, Adam B. Salmon, Daniel L. Smith, Jessica M. Snyder, David B. Lombard, Randy Strong

**Affiliations:** 1 Department of Pathology and Geriatrics Center, University of Michigan, Ann Arbor, Michigan, USA.; 2 The Jackson Laboratory, Bar Harbor, Maine, USA.; 3 Department of Epidemiology and Biostatistics, Indiana University School of Public Health, Bloomington, Indiana, USA.; 4 Department of Biological Sciences, Integrative Biosciences Center, Wayne State University, Detroit, Michigan, USA.; 5 Sam and Ann Barshop Institute for Longevity and Aging Studies and Departments of Physiology and Molecular Medicine, UT Health San Antonio, San Antonio, Texas, USA; South Texas Veterans Healthcare System, San Antonio, Texas, USA.; 6 Departments of Internal Medicine and Biostatistics, University of Michigan School of Medicine and School of Public Health, Ann Arbor, Michigan, USA.; 7 Department of Nutrition Sciences and Diabetes Research Center, University of Alabama at Birmingham, Birmingham, Alabama, USA; Birmingham VA Medical Center, Birmingham, Alabama, USA.; 8 Department of Internal Medicine, University of Michigan, Ann Arbor, Michigan, USA.; 9 Department of Psychiatry, University of Texas Health Science Center, San Antonio, Texas, USA.; 10 Department of Comparative Medicine, University of Washington, Seattle, Washington, USA.; 11 Division of Aging Biology, National Institute on Aging, Bethesda, Maryland, USA.; 12 Sam and Ann Barshop Institute for Longevity and Aging Research and Department of Cellular and Integrative Physiology, UT Health San Antonio, San Antonio, Texas, USA.; 13 Department of Nutrition Sciences and Nutrition Obesity Research Center, University of Alabama at Birmingham, Birmingham, Alabama, USA.

**Keywords:** Aging, Drug therapy, Glucose metabolism, Mouse models

## Abstract

Canagliflozin (Cana) is an FDA-approved diabetes drug that protects against cardiovascular and kidney diseases. It also inhibits the sodium glucose transporter 2 by blocking renal reuptake and intestinal absorption of glucose. In the context of the mouse Interventions Testing Program, genetically heterogeneous mice were given chow containing Cana at 180 ppm at 7 months of age until their death. Cana extended median survival of male mice by 14%. Cana also increased by 9% the age for 90th percentile survival, with parallel effects seen at each of 3 test sites. Neither the distribution of inferred cause of death nor incidental pathology findings at end-of-life necropsies were altered by Cana. Moreover, although no life span benefits were seen in female mice, Cana led to lower fasting glucose and improved glucose tolerance in both sexes, diminishing fat mass in females only. Therefore, the life span benefit of Cana is likely to reflect blunting of peak glucose levels, because similar longevity effects are seen in male mice given acarbose, a diabetes drug that blocks glucose surges through a distinct mechanism, i.e., slowing breakdown of carbohydrate in the intestine. Interventions that control daily peak glucose levels deserve attention as possible preventive medicines to protect from a wide range of late-life neoplastic and degenerative diseases.

## Abstract 2

The SGLT2 inhibitor canagliflozin extends median life span of male mice but does not increase life span of female mice.

## Graphical abstract

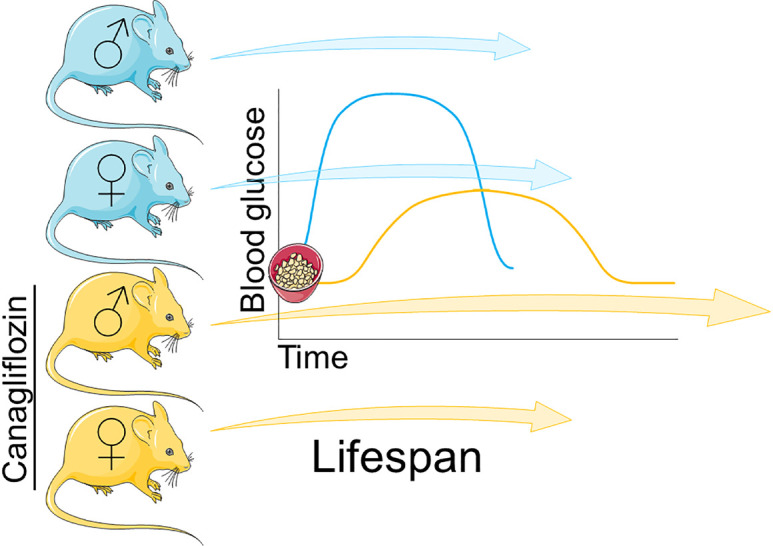

## Introduction

Aging is the dominant risk factor for most chronic diseases that afflict people in industrialized societies. There is now ample evidence, in mice, that the process of aging can be delayed or retarded by low-calorie diets (1–5), by natural or engineered mutations in any of several genes that modify growth hormone and IGF-1 signals (6, 7), and, more recently, by drugs added to food (8). Each of these approaches has been shown to extend life span and also to delay multiple forms of late-life illness, including both neoplastic and degenerative diseases. Identification of new drugs that extend mammalian life span is of great interest from at least 2 perspectives. First, elucidating the mechanism of action of such agents will provide new insights into the biology of the aging process and into the links between aging and multiple forms of late-life diseases. Second, these drugs may provide a starting point for development of antiaging interventions in humans.

The Interventions Testing Program (ITP), supported by the National Institute on Aging (NIA), tests the effects of drugs on mouse life span (http://www.nia.nih.gov/research/dab/interventions-testing-program-itp) (9, 10). ITP studies use annual cohorts of genetically heterogeneous mice (UM-HET3) equally distributed among 3 testing sites. Six compounds have thus far produced significant extension of life span in ITP studies in 1 or both sexes: rapamycin (11–13), 17α-estradiol (17aE2) (14, 15), nordihydroguaiaretic acid (NDGA) (15, 16), Protandim (14), glycine (17), and acarbose (15, 18). In a previous study, a seventh agent, aspirin, produced significant life span extension in male mice (16), but failed to show a benefit at 2 higher doses in a follow-up study (unpublished data).

The ITP experiment with Cana was motivated in part by our results using acarbose, a drug used in the treatment of type 2 diabetes (T2DM). Acarbose functions by inhibiting the breakdown of complex carbohydrates in the small intestine, thereby slowing the pace of glucose uptake and blunting the normal postprandial spike in blood glucose levels. In humans, acarbose treatment reduces glycated hemoglobin A1c (HbA1c) levels (19), although this effect was not seen in the ITP acarbose-treated mice (15). Acarbose, started at 4 months of age, led to a 22% increase in median life span in UM-HET3 male mice, and a significant but much smaller 5% increase in female mice (15). A test for effects on maximum life span (proportion alive at the 90th percentile) was significant in both sexes. When initiated at 16 months, acarbose leads to significant life span extension in male mice but a smaller effect in female mice (14). Acarbose-treated male mice also show a delay in age-dependent pathology in multiple systems, including inflammatory changes in the hypothalamus (20). Taken together, these results suggest that blunting of postprandial glucose peaks could delay aging and its consequences, including the illnesses that lead to death in mice. However, they do not exclude other possible mechanisms for acarbose action.

These results prompted us to consider whether other drugs that inhibit surges in postprandial blood glucose levels might also exert beneficial effects on longevity. Cana is an FDA-approved diabetes drug that acts primarily by inhibition of the sodium glucose transporter 2 (SGLT2) in the proximal tubule of the kidney (21). In nondiabetic individuals, SGLT2 is responsible for up to 97% of the reabsorption of the 160–180 g of glucose filtered by the kidney per day. To a lesser extent, Cana also inhibits SGLT1, a related glucose transporter with broader distribution, expressed in the small intestine, kidney, and heart, among other tissues. In a large group of patients with T2DM and elevated cardiovascular risk, Cana reduced death from cardiovascular causes, as well as nonfatal strokes, myocardial infarction, and heart failure (22). In patients with T2DM with kidney disease, Cana greatly reduced the risk of progression to kidney failure as well as the risk of cardiovascular events. Cana, like other SGLT2 inhibitors, reduces fasting blood glucose, HbA1c levels, and body weight, while modestly increasing blood ketone levels (23). Like acarbose, Cana suppresses the postprandial glucose surge (24, 25). The cardioprotective effects of SGLT2 inhibitors may be independent, at least partly, of their glycemic effects (26). Moreover, studies in mice have documented effects of Cana treatment on pathways relevant for life span: mTOR signaling, AMPK signaling, and FGF21 levels (27). In view of our previous results using acarbose, and the evidence for beneficial effects of SGLT2 inhibitors in humans, we conducted a test of the hypothesis that Cana would extend mouse life span.

## Results

### Survival.

**Figure 1:**
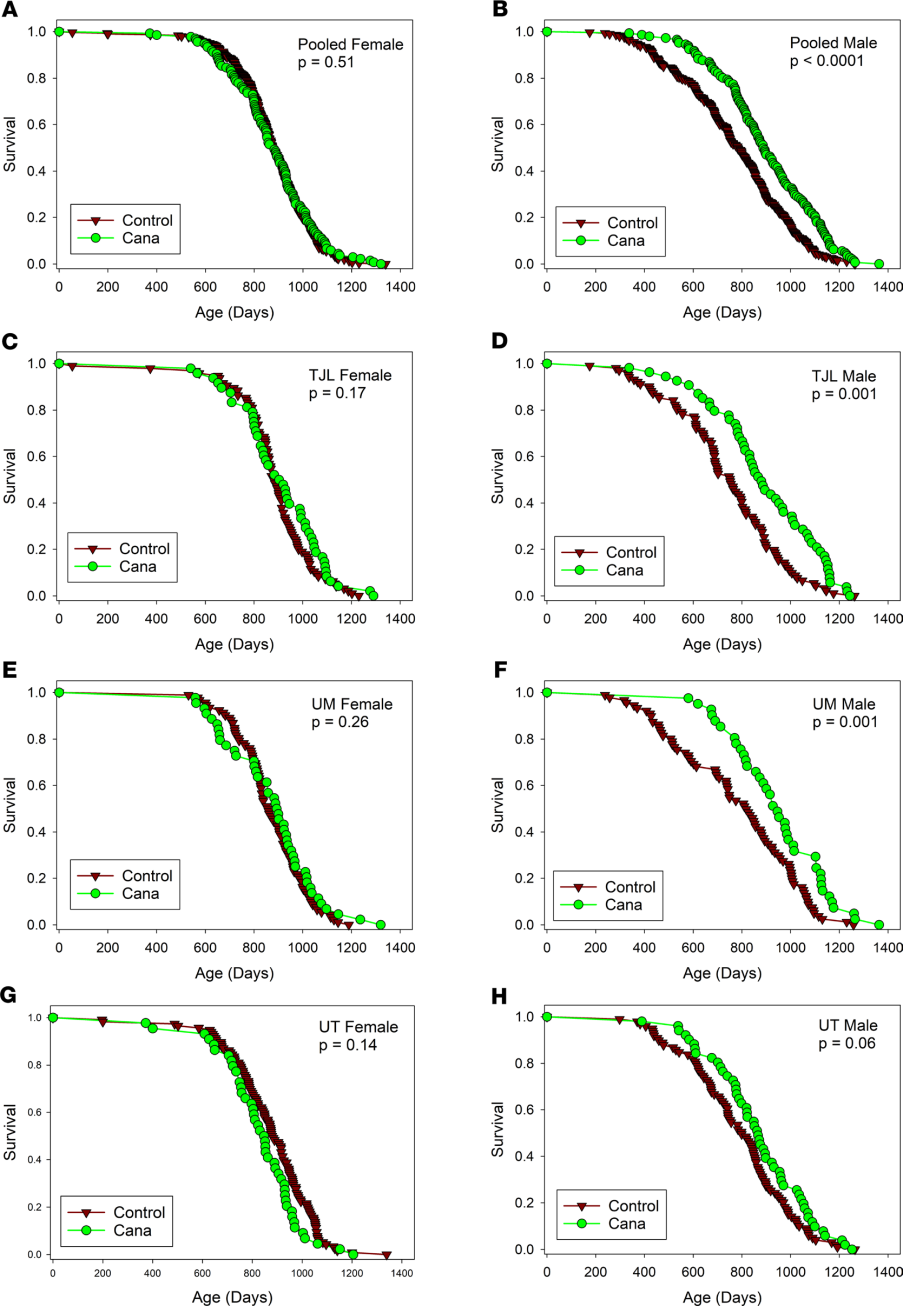
Survival curves for Cana-treated and sex-matched mice. (**A** and **B**) Data pooled across sites. (**C**–**H**) Site-specific results. Female mice are shown on the left, and male mice are shown on the right. *P* values show results of log-rank tests, not adjusted for multiple comparisons, with site as covariate for the pooled data sets (top row). There were no remaining live mice at the time of analysis. The number of mice in each group is included in the count column of Table 1.

In the context of the NIA ITP, groups of genetically heterogeneous (UM-HET3) mice born in 2016 were placed on a chow diet containing Cana at 180 ppm at the age of 7 months and followed for survival. Approximately 50 male mice and 50 female mice were exposed to Cana at each of the 3 test sites (The Jackson Laboratory [TJL], University of Michigan [UM], and University of Texas Health Science Center at San Antonio [UT]). Each site enrolled approximately 100 male and 100 female control mice, which also served as the control group for the other drugs tested in 2016. Date of death was recorded, and mice euthanized for humane reasons were considered to have died on the date of euthanasia. Figure 1 shows the survival curves for each combination of sex and site as well as the survival data pooled across all 3 sites. Table 1 shows the summary statistics for each site and for the pool. The prespecified primary outcome was the log-rank test for the pooled data set, calculated separately for each sex, and stratified for site within sex. For the C2016 cohort, there were no significant differences (log-rank test) among the 3 test sites for survival of male or female controls.

For male mice, Cana increased median survival age by 14%; the log-rank test yielded *P* < 0.001. The log-rank test was also calculated for each site separately, and despite much lower statistical power, survival was improved at each site, with *P* < 0.001 at TJL and UM and *P* = 0.06 at UT. Age at the 90th percentile survival point was taken as a prespecified index of long-term (“maximal”) survival, and this increased by 9% in the pooled male mice, with site-specific values of 15%, 9%, and 3% for TJL, UM, and UT, respectively. The Fisher’s exact test version of the Wang-Allison (WA) test (28) was used to evaluate the likelihood that the drug modified the proportion of survivors at the 90th percentile; the calculated *P* value, *P* < 0.001, suggests that Cana increases maximum survival in male mice. The WA statistic was significant at TJL and UM, but not at UT. None of these indices of improved survival were noted in female mice, for which the median survival age increased by only 1% (site-specific changes of 3%, 4%, and –4%), and age at the 90th percentile survival was increased by 1% (NS).

**Figure 2:**
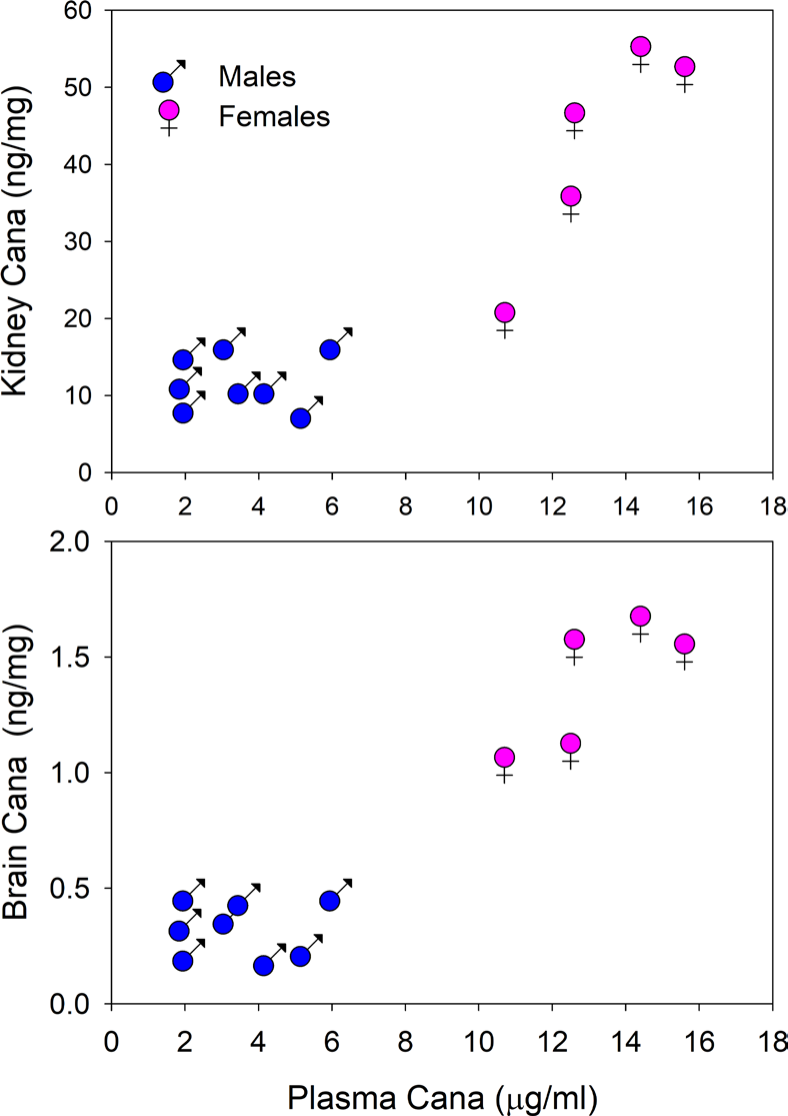
Cana levels in plasma, brain, and kidney of 22-month-old mice exposed to Cana from 7 months of age. Scatterplots show kidney levels of Cana (top) or brain levels (bottom) versus plasma levels. Each symbol refers to 1 mouse (5 female mice and 8 male mice were included).

We measured Cana levels in plasma, brain, and liver of mice aged 22 months, after exposure to Cana from 7 months of age. Plasma of female mice had a mean Cana concentration of 13.2 μg/mL (SEM = 0.9, *N* = 5), significantly higher than plasma levels of male mice (3.6 ± 0.6, *N* = 8; *P* < 0.001). Figure 2 shows that Cana levels in brain and in kidney were also significantly higher (*P* < 0.001) in female mice than in male mice. Plasma levels in brain and kidney were well correlated with levels in female mice but not in male mice. Plasma levels in young adults exposed to Cana for 8 weeks were lower than in mice exposed for 15 months but showed a similar sexual dimorphism, with values ranging from 2.12 to 3.53 μg/mL (*N* = 4) in female mice and from 0.36 to 0.86 μg/mL (*N* = 3) in male mice (*P* = 0.003 for the sex effect). These values are in the range of those reported for humans administered this drug (29). Thus, the absence of a survival benefit in Cana-treated female mice cannot be attributed to lower circulating levels of the drug. Husbandry technicians noted much higher urine output in the Cana-treated mice of both sexes throughout the study.

### Weight.

**Figure 3:**
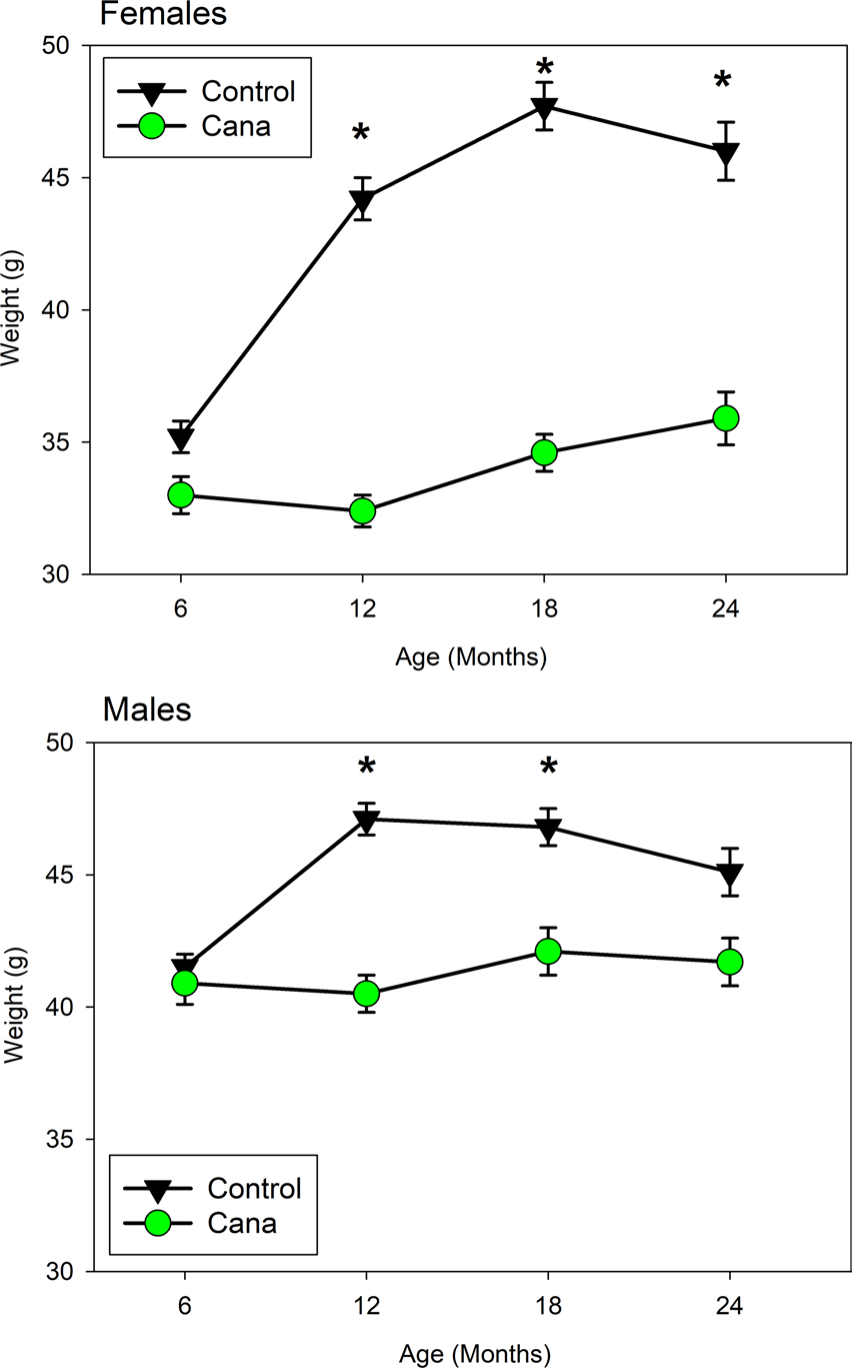
Mean weights for Cana and control mice, ages 6–24 months. Values are mean levels for live mice, separately for each sex, pooled across sites. Cana treatment was initiated at 7 months. *P < 0.0002 for drug effect at ages 12–24 for female mice and 12–18 for male mice. *N* declined with age from 151–116 for Cana-treated male mice and from 136–107 for Cana-treated female mice, with approximately 2-fold higher numbers of control mice.

Cana limits weight gain in both sexes, as shown in Figure 3. In female mice, the effect on weight is dramatic (17%–19%) and statistically significant (*P* < 0.0001) at each age after initiation of drug treatment at 7 months. Cana-treated male mice are 5%–8% lighter than controls at ages 12 and 18 months (*P* < 0.0002). Mean weight of control male mice declines between 18 and 24 months, presumably related to onset of late-life illness and/or death of the heaviest male mice, but this effect is blunted in Cana-treated mice. The male-specific alteration in life span is apparently not attributable to a male-specific blockage of midlife weight gain.

### HbA1c levels.

HbA1c was evaluated in whole blood of 48 female and 45 male mice, after exposure to Cana for 7–8 months starting at 7 months of age, with samples taken at all 3 test sites. There was no effect of Cana on HbA1c levels (control male mice 3.8%, Cana-treated male mice 3.7; control female mice 3.5, Cana-treated female mice 3.5), although the difference between sexes was significant at *P* < 0.0001 in a 2-way ANOVA. This result is consistent with our previous data on HbA1c in acarbose-treated mice (15), although Cana diminishes HbA1c in patients with diabetes (22).

### Body composition and glucose homeostasis.

**Figure 4:**
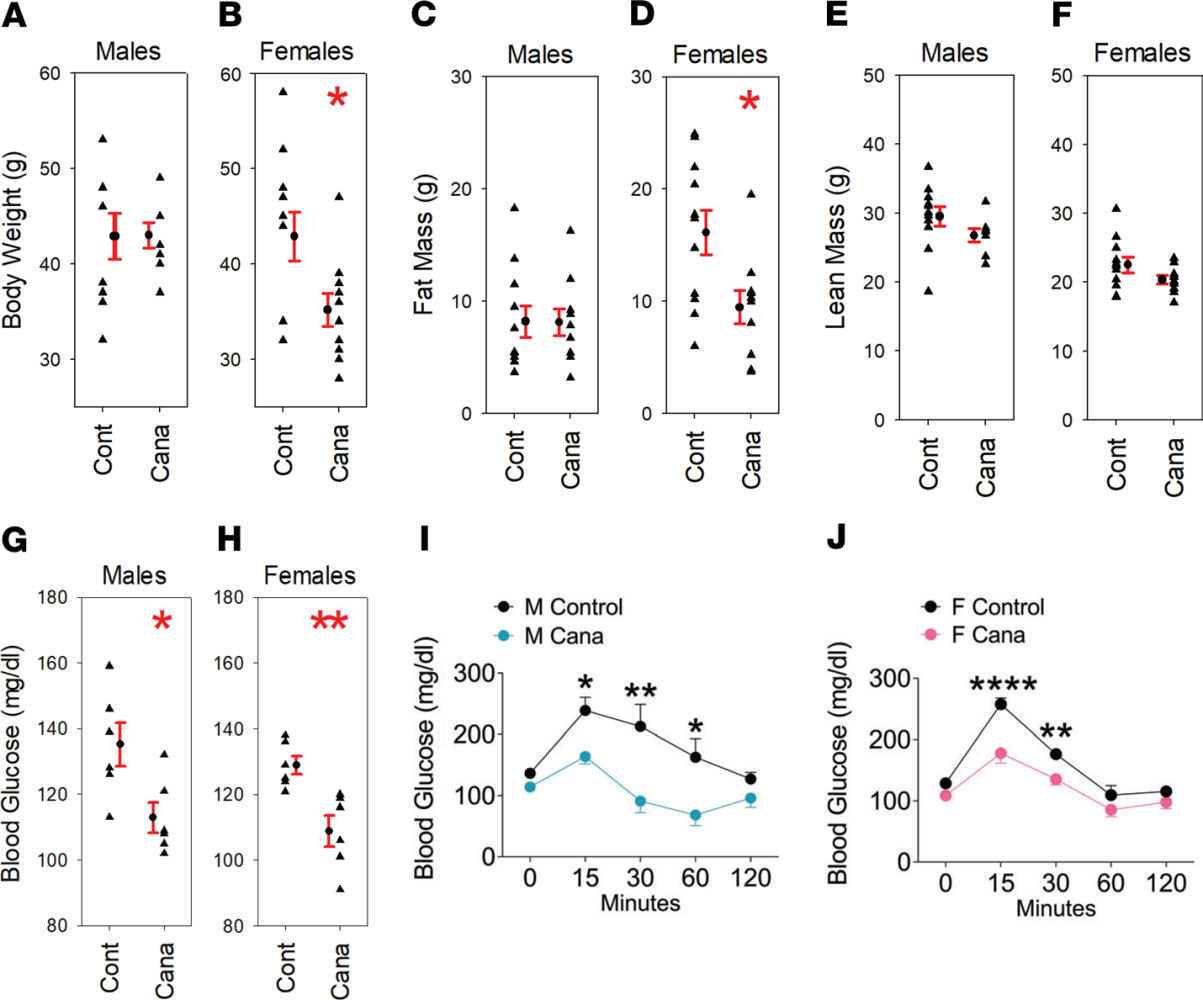
Body composition and glucose homeostasis in Cana versus control mice. Each symbol represents a different mouse, with mean ± SEM also shown. Mice were given Cana from 7 months of age, and tested when 20–22 months old. (**A**–**F**, **I**, and **J**) The body composition and glucose tolerance data were obtained using 8–11 mice of each sex for each group, and (**G** and **H**) the fasting glucose data were obtained from 6 mice of each sex for each group. The glucose tolerance test data show results for 5 male mice and 6 female mice in each treatment group. *P < 0.05, **P < 0.01, ****P < 0.0001.

UM-HET3 mice born at UM received Cana or control chow from the age of 7 months and were then transferred at 20–22 months of age to the Sadagurski laboratory at Wayne State University for metabolic testing and evaluation of body composition. Figure 4 shows that Cana did not alter total mass, lean mass, or fat mass in male mice but led to a significant (18%) decline in total mass and a 41% decline in fat mass in female mice. Glucose, measured after a 3- to 4-hour fast, was lower in the Cana-treated mice than in controls for both sexes. An i.p. glucose tolerance test showed significantly better responses in Cana-treated mice of both sexes.

### Cause of death.

All mice dying at ages above 600 days were fixed, and a random sample (10 male mice and 10 female mice from each site in each of 2 drug conditions) was sent for evaluation to the University of Washington Geropathology Research Program with no information regarding Cana status. The mean age of Cana-treated mice was 873 days (male mice) or 892 days (female mice), and the values for control mice were 845 and 857 for male and female mice, respectively. Each case was evaluated in-house by the same board-certified veterinary pathologist, who attempted to infer the cause(s) of death, i.e., the lesion(s) that appeared most likely to have led either to the death of the mouse or the decision to euthanize the mouse for humane reasons at the end of life.

Table 2 shows the distribution of inferred causes of death (CODs) among the mice. Statistical power is poor, because most individual diagnoses appeared in 7 or fewer of the 120 cases. The most common lethal lesion was hematopoietic neoplasia (HPN), a collection of hematopoietic neoplastic diseases, including lymphoma and histiocytic sarcoma, that are difficult to distinguish without special stains or molecular characterization. HPN was noted in 18 of 60 Cana-treated mice and in 16 of 60 controls. For most lesions, there were no effects of site (although as noted with very low statistical power), but HPN as a (COD) was more common at TJL (16 of 28 cases in which a COD could be inferred) than at UM and UT (18 of 70 nonautolyzed cases, *P* = 0.003). None of the classes of lethal illness shows a statistically significant difference between treated and control mice. For Cana-treated mice, there were 45 cases for which a single COD could be assigned; this excludes the 10 cases where multiple lesions were deemed contributory and the 5 cases with advanced autolysis. Of these 45 mice, 34 (76%) died of some form of neoplastic disease. Similarly, 36 of the 44 evaluable control cases (82%) died of neoplasia (*P* = 0.12 for contrast with Cana-treated mice.)

### Incidental pathology at end of life.

In addition to attempting to infer a likely COD, the pathologist recorded presence or absence of lesions in multiple tissues and assigned a grade of severity for a separate set of lesions (see Supplemental Table 1; supplemental material available online with this article; https://doi.org/10.1172/jci.insight.140019DS1). Supplemental Table 1 shows 21 lesions (in adrenal, atrium, lung, heart, liver, kidney, mammary gland, thalamus, pancreas, spleen, and thyroid) that were scored as present or absent, with *P* values showing results of the Fisher’s exact test comparing Cana to control mice. Drug treatment had no significant effects on frequency of any of these lesions. Supplemental Table 2 shows 14 lesions that were graded on scales of 0–2 or 0–4, depending on the lesion. The *P* value shown reflects the 2-sided Student’s *t* test contrasting treated and control mice. Only 1 form of pathology met the criteria for statistical significance: the mean level of liver telangiectasia/angiectasis was 0.41 for Cana and 0.09 for control mice (*P* = 0.01). Since this lesion is graded on a scale of 1–4, with a score of 1 corresponding to minimal change, it seems unlikely that this drug effect has any clinical implication. With that exception, the data suggest that Cana-treated mice show similar incidence and severity of most forms of end-of-life pathology.

## Discussion

Interventions, whether diets, mutations, or drugs, that slow aging and postpone multiple forms of late-life illness provide valuable tools for investigation of the cellular and molecular mechanisms for aging, as well as hints as to where those tools might most usefully be applied. Among the 7 ITP-tested drugs that have shown significant longevity benefit, Cana, acarbose, 17aE2, and rapamycin have the largest effect on median life span, and each of these also has a significant effect on our measure of maximum life span (28), in one or both sexes. Surprisingly, acarbose, 17aE2, and rapamycin can each produce significant life span extension even when started at ages 60%–70% of the median survival age of controls, i.e., 16–20 months of age (13, 14) (unpublished observations for 17aE2). Life span studies in which Cana is initiated at older ages are now under way at each ITP site. Terminal (end-of-life) necropsies for each of these 4 drugs, including the data on Cana shown here, lead to the same conclusion: the spectrum of lethal and nonlethal late-life illnesses is not changed in quality or severity, even though the treated mice are older at time of death. Although this conclusion is limited by the low statistical power of small case series, the implication is that the drugs extend healthy life span, i.e., produce longer life span by delaying the onset or progression of the diseases most likely to lead to death or terminal morbidity, most of which are neoplastic disease in UM-HET3 and most other mouse stocks. A comprehensive series of cross-sectional necropsies of rapamycin-treated mice (30), euthanized at 22 months of age, supported this inference by showing 9 varieties of age-dependent lesions, in multiple organs, the incidence of which was reduced by rapamycin.

The results of these studies show an unexpected degree of sexual dimorphism. The percentage benefit of rapamycin, at each tested dose, is higher in female mice than in male mice, although this seems likely to reflect the higher blood rapamycin levels, in female mice, at matched doses in food (11). It is unclear which sex would show preferential benefit at fixed blood levels of rapamycin. Acarbose, both in the original report and in an independent follow-up study (15, 18), led to significant life span extension in both sexes, but with much larger effects in male mice. The nonfeminizing steroid 17aE2, in contrast, produced benefits only in male mice; 17aE2-treated male mice lived significantly longer than female mice whether or not the female mice had received this agent (14, 15). Two other tested drugs also produced significant life span benefits in male mice only. NDGA, an antioxidant with antiinflammatory properties, led to male-specific life span increase of up to 12% (*P* = 0.006 by log-rank test), although without a significant increase in maximal life span; the benefit, and its limitation to male mice, was replicated in an independent study (14–16). Protandim, a mixture of botanical extracts known to activate the Nrf2 stress-response pathway, also led to increased life span in male mice only (14). Only one other tested agent, the amino acid glycine, produced significant life span extension in both male and female mice, and the effect, though seen at each of the 3 ITP test sites, was quite small, yielding less than a 5% increase in median longevity (17). Effects of acarbose, including an increase in glucose tolerance and mTORC2 function (31) and a reduction of subscapular fat depots (18), were seen principally in male mice, and the effects of 17aE2 on metabolite profiles, glucose control, grip strength, and rotarod performance were also male-specific (32). Both acarbose and 17aE2 reduce mid-life hypothalamic inflammation in male mice only (20). More work will be needed to work out the molecular and physiological explanation(s) for these sex-specific effects, but it is notable that at least some of these effects are blocked by castration of young adult male mice before the initiation of treatment by acarbose or 17aE2 (31, 32), suggesting that levels of male hormones, even in postpubertal adults, sensitize mice to life span extension by these drugs.

Cana and acarbose each reduce serum glucose in mice, or people, who have recently consumed a carbohydrate-rich meal, but they do so through partly different mechanisms. In addition to potently inhibiting SGLT2, Cana also reduces glucose uptake via SGLT1 in the small intestine and kidney, albeit with less potency than its effect on SGLT2 (33, 34). Physiologically, Cana suppresses glucose reuptake in the kidney, as well as delaying glucose absorption in the small intestine (21). Acarbose inhibits breakdown of polysaccharides to absorbable monosaccharides in the intestine. These observations thus suggest strongly that the life span benefits of each drug, and presumably the other benefits already shown in acarbose-treated mice, are a consequence of lower maximal postprandial glucose levels. Metabolically, Cana and other SGLT2 inhibitors (SGLT2i) lower body weight, HbA1c, and peak glucose levels in humans; improve insulin resistance; increase serum ketones; enhance fatty acid oxidation; and elevate β cell function (21). In our mice, Cana led to lower fasting glucose and improved glucose tolerance in both sexes, and to lower fat mass in female mice only. Neither Cana nor acarbose, at the doses used by the ITP, seems to diminish HbA1c, suggesting that the life span and health benefits in mice may depend more on peak glucose levels than on average glucose levels integrated over the day. UM-HET3 mice do not spontaneously develop diabetes, and Cana reduces the elevated HbA1c levels observed in diabetic rats and mice (33, 35, 36). Thus, it is possible that our studies in UM-HET3 mice underestimate the potential health benefits of long-term Cana treatment in humans, in which glucose intolerance and frank diabetes are common features of aging (37). The dramatic effects on weight shown in both male and female mice in Figure 3, and the data on fasting glucose and glucose tolerance included in Figure 4, show that Cana has strong effects on fuel use and metabolic partitioning in both sexes. However, additional studies will be needed to evaluate sex-specific modulations of relevant factors, particularly including insulin production and sensitivity, fat cell homeostasis and adipokine production, and hypothalamic and gastrointestinal circuits that modulate appetite and satiety. Notably, no major sex-specific effects of Cana have been reported in clinical trials documenting its beneficial effects in humans. However, our results are consistent with prior studies of long-term Cana administration in rats (38). In these studies, Cana, in a dose-dependent manner, extended rat life span, with a much greater effect observed in male mice than in female mice. As with other ITP drugs for which sexual dimorphic benefits are observed, the basis for the sex specificity of Cana on rodent life span remains unclear.

The end-of-life necropsy data for Cana-treated mice are consistent with previous findings on mice in which their life span was extended by 17aE2, acarbose, and rapamycin (12, 15). Specifically, the spectrum of lethal and nonlethal lesions, with a few exceptions, is similar between control and drug-treated mice, but delayed in timing. We see 2 complementary implications, one drawn from pathophysiology and the other from biogerontology. From the first perspective, our results justify follow-up studies to learn more about how these drug-induced changes, such as the blunting of peak glucose levels that accompanies acarbose and Cana treatment, might delay or decelerate the most frequent forms of age-related illnesses, such as neoplasms of many tissues and the wide range of degenerative diseases seen in mice and humans, and can begin to test dietary and pharmacological interventions aimed at controlling glucose peaks. In parallel, researchers focused on questions in the basic biology of aging can use these drugs as experimental tools for testing hypotheses about how aging might be linked upstream to glucose or mTOR or steroid balance, and downstream to disability and dysfunction.

Cana and other SGLT2i show a wide spectrum of beneficial effects in humans and rodent models, which in principle could contribute to extension of mouse life span by Cana. The basis for these protective effects is not completely understood, and may not directly reflect the effects of Cana on glucose homeostasis. For example, Cana reduces systolic and diastolic blood pressure, fatal and nonfatal cardiovascular events, and hospitalization for heart failure (22). The potent cardioprotective effects of Cana and other SGLT2i are not observed to the same degree in patients treated with other, comparably potent, glucose-lowering agents, suggesting that pleiotropic effects of SGLT2i may be involved (23). Since most UM-HET3 mice die of neoplastic disease, the effects of Cana on cancer may be particularly relevant for its ability to extend mouse life span. In preclinical studies, Cana administered at high doses to rats increased the frequency of adrenal and renal tubular tumors, and induced Leydig cell hyperplasia and adenomas at all doses in male mice (38). In a recent meta-analysis, however, SGTL2i use in humans was not associated with an overall increased cancer risk, and Cana specifically was associated with a reduced risk of gastrointestinal cancer (39).

Hypothetically, Cana might reduce mortality associated with neoplasia in mice by modulating glucose metabolism in tumor cells. The metabolic requirements of rapidly dividing cancer cells are quite distinct from those of normal cells, most of which are ordinarily nonmitotic. In contrast, cancer cells must constantly generate large quantities of new biomolecules for macromolecular synthesis and cell replication. For most cancer types, glucose, and particularly glycolytic intermediates derived from it, represents a critically important fuel (40). SGLT1 and SGLT2 are functionally expressed on human prostate and pancreas cancers, and Cana treatment of a pancreas cancer xenograft potentiated the effects of genotoxic chemotherapy (41). Cana inhibits growth of SGLT2-expressing hepatocellular cancer xenografts via inhibition of glycolysis and suppression of tumor angiogenesis (42), and by reducing β-catenin expression (43). In a mouse model of nonalcoholic steatohepatitis-induced hepatocellular carcinoma, Cana reduced hepatic triglyceride accumulation, inflammation, and tumorigenesis (44). Cana has also been proposed to suppress growth of prostate and lung cancer cells via a non-SGLT1/2-dependent mechanism involving inhibition of mitochondrial Complex I (45). Thus, Cana treatment may be acting, in part, by suppressing initiation and/or progression of age-associated cancers in UM-HET3 mice, through SGLT1/2-dependent or -independent mechanisms, an idea to be explored in follow-up studies.

Osataphan et al. described numerous effects of Cana on whole-body metabolism and signaling pathways in mice that may contribute to its beneficial effects on life span (27). In mice fed a high-fat diet to induce obesity, Cana reduced weight gain and blood glucose levels; and in mice fed either high-fat or normal chow, Cana enhanced fat and ketone body metabolism at the expense of glucose utilization. Notably, the use of alternative fuel sources such as fatty acids and ketones has been linked to the pro-longevity intervention calorie restriction (46). Cana suppressed hepatic TORC1 signaling, while increasing AMPK activity in this tissue; both effects are associated with life span extension in mice and other organisms (47). Cana treatment also increased serum levels of FGF21, a peptide hormone that induces a shift from carbohydrate to lipid metabolism; FGF21 extends life span in mice when overexpressed (48). Since only male mice were examined in the Osataphan study (27), it is impossible to know whether any of these Cana effects might show sexual dimorphism. Such data might help shed light on which among the pleiotropic effects of Cana might be most relevant in promoting longer life, since the longevity benefit of Cana is observed in male but not female mice.

Combining COD and incidental lesion scores is a powerful approach to evaluate the effects of a drug on progression of age-related pathology over a life span. Male mice given Cana showed an increase in life span compared with controls, but with similar COD and incidental lesion scores, suggesting a delay in major and incidental lesion pathology associated with aging. A cross-sectional study, now under way, will produce specimens for histopathology study at a defined age, 22 months, at which the complications of selective death and variable postmortem interval have been greatly reduced. Such cross-sectional designs do not provide useful insights into possible drug effects on distribution of COD diagnoses, but can provide a more finely grained look at drug effects on age-dependent changes in multiple tissues and organs (30). COD and incidental lesion scores can be used as a framework for establishing pathological endpoints (49) and can be combined with physiological performance tests to further validate the effectiveness and safety of Cana or other drugs, either alone or in combination with other previously validated antiaging drugs.

Prospective clinical trials for Cana effects in humans might be enriched by inclusion of endpoints that are signals of age-dependent diseases and physiological change that are nominally independent from the effects of the diseases, such as diabetes, for which the drug is prescribed. The dose used in this study provides 6 mg of drug per kilogram of mouse body weight per day, similar to the human therapeutic dose of 100–300 mg/d for a 70 kg person. In parallel, secondary analysis of databases that include long-term follow-up on patients who are receiving Cana therapeutically might help to test the idea that this agent retards clinically important aspects of multisystem aging in men or women or both. The ITP results using both Cana and acarbose suggest that many aspects of age-related pathology, including those that lead to death in mice, could be sensitive to modulators of peak glucose levels, rather than integrated measures of average glucose values, with implications for both dietary and pharmacological avenues for prevention of late-life diseases in humans. Delineation of the mechanisms connecting Cana to slower aging, whether by effects on glucose or potentially through other pathways, are bound to have implications for our understanding of both mammalian aging and organ-specific pathophysiology.

## Methods

### Mice.

These experiments used mice of the UM-HET3 genetically heterogeneous stock, produced by mating CByB6F1 mothers (JAX stock 100009), whose female parents are BALB/cByJ and whose male parents are C57BL/6J, to C3D2F1 fathers (JAX stock 100004), whose mothers are C3H/HeJ and whose fathers are DBA/2J. Each UM-HET3 mouse is genetically unique, but each shares 50% of its genetic endowment with every other UM-HET3 mouse, and the population thus consists of full sibs with respect to nuclear genes. Mice from second and subsequent litters were weaned into cages containing 3 male or 4 female mice and assigned to a control or treatment (Cana) group using a random number table. Mice at each test site were produced over a period of 6 months in 2016 in roughly equal monthly cohorts. The C2016 cohort also included mice treated with other agents, and the results of those studies will be reported separately. Mice in the Cana group received this agent at 180 mg/kg of chow, from 7 months of age; this dose is equivalent to 30 mg/kg mouse body weight for a 30 g mouse eating 5 g of food each day. Mice were weighed at 6, 12, 18, and 24 months but not subjected to any other manipulation. Cages were inspected daily, and deaths were noted. Mice that were deemed unlikely to live for more than another 24 hours, based on a symptom checklist, were euthanized for humane reasons, with the day of euthanasia taken as the best estimate of the date of natural death for statistical purposes. Mice removed from the study, either for fighting or for other technical reasons (e.g., chip ID dysfunction, escape, accidental injury), were treated as known to be alive on the day of removal but lost to follow-up at that point. These “removed” mice are included in the Kaplan-Meier survival calculations but not in calculations of median or 90th percentile survival age. Removed mice made up 4.8% of the original control group, and 3.8% of the Cana group. No other mice were censored from analyses. Details of husbandry conditions, such as cage changes, base diet, temperature, and light/dark cycle have been described extensively in other ITP reports (11, 14). Specific pathogen–free status is assessed quarterly at each site using a combination of serological and molecular methods, and all such tests have been negative for each colony throughout the period reported.

### Pathology.

Each mouse found dead, or euthanized for humane reasons, was fixed as soon as possible. Incisions were made in the cranium, thorax, and abdomen, and the specimen then immersed in 10% neutral buffered formalin for storage at room temperature. Random samples from each of the 3 sites were later sent to the University of Washington Geropathology Research Program for gross inspection and for preparation of slides for histological examination. During gross examination and dissection, all obvious external and internal abnormalities were recorded. Tissues evaluated and collected for histological examination included decalcified cross section of skull with brain (when intact), lung, mediastinal lymph node and fat, thymus (when able to identify), heart, kidney, adrenal glands, liver, spleen, pancreas, mesenteric fat and lymph node(s), thyroid gland, salivary glands, reproductive tract (consisting of uterus and ovaries for female mice and testis, epididymis, and seminal vesicles for male mice), and any additional lesions noted grossly. The gastrointestinal tract was inspected grossly but not routinely examined histologically owing to autolysis. Tissues were routinely processed and paraffin embedded, after which 4–5 μ sections were stained with H&E for histological assessment.

Tissues were examined for a single major abnormality such as a malignant tumor extensively involving an organ or involving multiple organs, severe inflammatory disease, or an end-stage degenerative process such as cardiomyopathy or glomerulonephropathy. If such a process was identified, then this was considered the COD. In some mice, more than 1 severe abnormality was identified, and in these cases, death was attributed to 2 or more distinct lesions; these are listed in Results as “multiple” COD cases. If the carcass was in generally good postmortem condition but no obvious COD was identified, the COD was listed as “unknown.” If autolysis precluded critical histological evaluation of tissues for COD, a designation of “autolysis” was made. Some incidental lesions were recorded as present or absent, and other incidental lesions were graded with a severity score.

### HbA1C.

Male and female mice at age 7 months were fed control or Cana diets for 7–8 months. These mice were not involved in the life span protocol. Blood was collected into EDTA-coated tubes from mice that were fasted for 6 hours starting at 8–9 am. Samples were shipped to UT, and HbA1c percentage for each whole blood sample was measured by immunoassay using a DCA Vantage Analyzer (Siemens Healthineers) according to the manufacturer’s instructions.

### Measurement of Cana in tissues.

The liquid chromatography–tandem mass spectrometry system consisted of a Shimadzu SIL 20A HT autosampler, LC-20AD pumps, and an AB Sciex API 4000 tandem mass spectrometer for plasma and an AB Sciex API 3200 tandem mass spectrometer for brain or kidney, both with turbo ion spray. The LC analytical column was an ACE Excel C18-PFP (75 × 3.0 mm, 3 μ) purchased from Mac-Mod Analytical, maintained at 25^o^C. Mobile phase A contained 0.1% formic acid dissolved in water. Mobile phase B contained 0.1% formic acid dissolved in 100% HPLC-grade acetonitrile. The flow rate of the mobile phase was 0.4 mL/min. Standard curves used Cana (MilliporeSigma). For plasma, calibrator samples used concentrations from 0.1–5 mg/mL, with Lidocaine D10 as an internal standard. The ratios of Cana to lidocaine D10 peak areas for each unknown sample were compared against a linear regression of the ratios obtained by the calibration samples to quantify Cana. The Cana transition was detected in positive mode at 445.3/267.2 Da. The Lidocaine D10 transition was detected at 245/96 Da. For kidney and brain, standard curves used a mixture of male and female control kidney homogenate spiked with Cana over a range of 0.01–20 mg/mL, and the Cana expressed as nanograms per milligrams of tissue.

The assessment of food levels included measures of within- and between-pellet homogeneity and drug stability. Mean levels in prepared food averaged 85% of expected (nominal) concentration, with a range of 53%–180% of expected over 11 batches. Within- and between-pellet heterogeneity averaged 8%.

### Metabolic analyses.

Lean and fat body mass were assessed by a Bruker Minispec LF 90II NMR-based device. An i.p. GTT was performed on mice fasted for 3–4 hours. Animals were injected i.p. with D-glucose (2 g/kg) and blood glucose levels were measured at the indicated time points, with the preinjection blood sample used as a measure of fasting glucose.

### Statistics.

The analytical plan was specified before the initiation of the study. Survival was evaluated using the log-rank test, stratified by site, conducted separately for male and female mice. *P* values are reported without adjustment for multiple comparisons. Secondary analyses of survival were conducted on data from each of the 3 test sites. The WA test (28) was used to assess an analog of “maximal life span.” This method uses the Fisher’s exact test to contrast the proportions of mice alive or dead at the 90th percentile age of the joint (shared) survival table for control and Cana-treated mice together. The primary test employed the sum of live and dead mice taken from each test site, separately for each sex, using the 90th percentile age for that site and sex. Drug effects on body weight were assessed by the 2-tailed Student’s *t* test, separately at each age for each sex, pooling across sites. Drug effects on specific inferred cause(s) of death, and on lesions scored as present or absent, were evaluated using the Fisher’s exact test, pooling across site and sex. Drug effects on lesions that were graded were assessed using the 2-tailed Student’s *t* test. HbA1C results were analyzed by 2-way ANOVA, with sex and drug (Cana) as the predictor variables. At time of publication, all survival, weight, and metabolic data reported in this paper will be made available in the Mouse Phenome Database (https://phenome.jax.org/projects/ITP1).

### Study approval.

All animal experiments were approved by the IACUC of TJL, UM, and UT.

## Author contributions

DEH, RS, and RAM supervised the project design, technical personnel, interpretation of results, and preparation of manuscript drafts. RAM and DL wrote the first draft of the paper. JMS and WCL conducted the pathology analysis and interpreted the pathology findings. AG, NK, and MB participated in the statistical analysis. DLS, DBA, and WTG first suggested inclusion of Cana in the ITP study. MAJ carried out the pharmacological testing. MS, LD, and HJ conducted the body composition and glucose tolerance studies. VD and EF supervised the husbandry work at UT. PR supervised the husbandry work at TJL. NR and JN provided advice on experimental design and interpretation. FM served as project officer for the NIA and contributed to program development, experimental design, and interpretation.

## Supplementary Material

supplemental data

## Figures and Tables

**Table 1:**
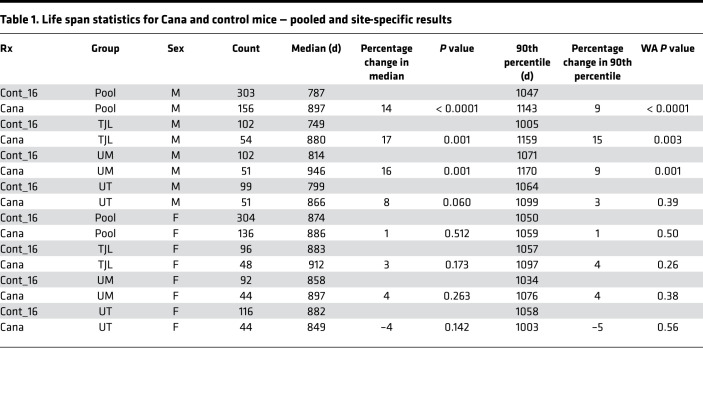
Life span statistics for Cana and control mice — pooled and site-specific results

**Table 2:**
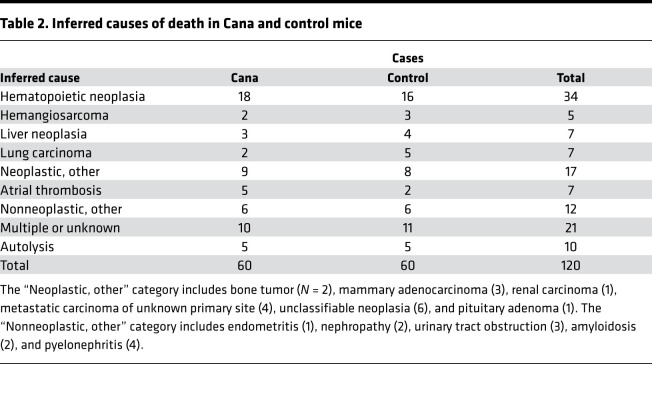
Inferred causes of death in Cana and control mice

## Version 1 09/29/2020

In-Press Preview

## Version 2 11/05/2020

Electronic publication
