# Supplementary material for: Canagliflozin extends life span in genetically heterogeneous male but not female mice
Source: JCI Insight. 2020 Nov 5;5(21):e140019. doi: 10.1172/jci.insight.140019 (PMC7710304; doi:10.1172/jci.insight.140019)
Supplement: supplemental data [file jciinsight-5-140019-s157.pdf]

Supplemental Table 1: Incidental Pathology Findings – Lesion Counts

Lesion	Cana Cases	Control Cases	p-value
Adrenal subcapsular cell hyperplasia	35	37	0.85
Adrenal neoplasia	9	3	0.12
Atrial thrombus	11	5	0.12
Eosinophilic crystalline pneumonia	5	7	0.76
Hemangiosarcoma	6	6	0.99
Heart neoplasia (non-HPN)	2	5	0.27
Hematopoietic malignancy (HPN)	22	20	0.85
Hepatocellular hypertrophy/hyperplasia	21	15	0.32
Hyaline droplets (kidney tubules)	10	7	0.60
Hepatocellular adenoma	2	5	0.27
Mammary adenocarcinoma or adenoma	6	6	0.99
Thalamic mineralization	12	14	0.82
Pancreatic inflammation	5	2	0.27
Pulmonary adenoma	17	10	0.19
Pulmonary carcinoma	9	15	0.18
Pulmonary neoplasia (other)	5	5	0.99
Extra-medullary hematopoiesis (spleen)	19	12	0.27
Splenic neoplasia	12	19	0.21
Thyroid follicular dilation or cystic follicles	41	36	0.34
Thyroid follicular hyperplasia	8	6	0.59

The tabulated values are the number of cases in which the lesion was observed, in a collection of 58 Cana and 58 Control mice (four mice were not evaluable, due to advanced autolysis.) The p-value reflects the two-tailed Fisher Exact Test. These counts are underestimates, since autolysis will have rendered many lesions undetectable, but the extent of autolysis was not significantly different ($p = 0.7$) for Cana and Control mice.

Supplemental Table 2: Incidental Pathology Findings: Graded Lesions

Lesion	Cana	Control	p-value
Adrenal degeneration (*)	1.76	1.90	0.55
Cardiomyopathy	1.46	1.65	0.26
Glomerulonephropathy	1.55	1.85	0.12
Liver degeneration/necrosis	1.06	0.64	0.10
Liver telangiectasia/angiectasis	0.41	0.09	0.01
Macrovesicular hepatic lipidosis	0.36	0.46	0.58
Microvesicular hepatic lipidosis	0.81	0.89	0.70
Ovarian degeneration (*)	3.67	3.42	0.26
Pancreatic islet hyperplasia	0.76	1.06	0.13
Pancreatic exocrine atrophy	1.09	0.76	0.39
Salivary gland lymphoid aggregates	1.29	1.30	0.99
Testicular degeneration (*)	1.48	1.83	0.20
Uterine cystic endometrial hyperplasia	2.33	2.10	0.41

Lesions were graded on scales (0 – 2 or 0 – 4, depending on lesion). Values shown are mean levels in Cana and Control mice, shown with the p-value from a two-sided Student's t-test (equal variance assumption). The number of scorable cases varied among lesions, because autolysis renders some lesions unscorable while allowing others to be assigned a grade.

*Scoring for adrenal gland, ovary, and testes includes assessment of a number of age-related lesions in these organs, including adrenal pigment and cortical changes; ovarian atrophy, interstitial cell hyperplasia, and pigment; and testicular atrophy and mineralization.
